# Dexmetomidine, fentanyl and esmolol on prevention of the hemodynamic effects of laryngoscopy and endotracheal intubation. a prospective, randomized, double-blind study

**DOI:** 10.1186/2197-425X-3-S1-A210

**Published:** 2015-10-01

**Authors:** M Mavri, K Rellos, I Pantazopoulos, C Kampolis, I Floros, N Iacovidou, T Xanthos, C Pantazopoulos

**Affiliations:** Central Clinic of Athens, Research and Treatment Medical Center, Anaesthesiology and Intensive Care, Athens, Greece; Evaggelismos General Hospital of Athens, Intensive Care Unit, Athens, Greece; Laiko General Hospital of Athens, University of Athens, Pathophysiology Clinic, Athens, Greece; Laiko General Hospital of Athens, University of Athens, Intensive Care Unit, Athens, Greece; University of Athens, Medical School, Neonatology, Athens, Greece; University of Athens, Medical School, MSc Cardiopulmonary Resuscitation, Athens, Greece

## Intr

The hemodynamic effects of laryngoscopy and endotracheal intubation are well known. A variety of drugs, like fentanyl, esmolol and lately dexmetomidine, have been used in order to attenuate the stress response to laryngoscopy and endotracheal intubation. Esmolol is an ultrashort acting beta 1 selective adrenergic antagonist, while dexmetomidine is an alpha 2 adrenergic receptor agonist. Fentanyl is a short-acting opioid.

## Objectives

The scientific objective of this study, was to compare the efficacy of dexmetomidine, fentanyl and esmolol for attenuation of sympathetic response during laryngoscopy and endotracheal intubation.

## Methods

The present study included sixty (60) elective surgery patients with ASA 1 and 2, aged 20-60 years, who needed endotracheal intubation. Patients were randomly allocated into 3 groups. Each group included twenty patients (n = 20). Patients in group D (Dexmetomidine) received 1mcg/kg dexmetomidine with infusion in 10 min, patients in group F (Fentanyl) received 2 mcg/kg fentanyl, while in group E (Esmolol), patients received 2 mg/kg esmolol two min before induction. All patients had Mallampati and Cormack-Lehane Grades 1 or 2. All patients were intubated in less than 30 sec. Systolic, diastolic, mean arterial pressures and heart rates were measured before induction, before intubation and 1, 2, 4 and 10 min after intubation.

## Results

When recorded values before induction and intubation were compared to the measurements of the groups, it was found that 4 and 10 min after intubation, heart rate in group D and systolic, diastolic and mean arterial pressures in group E, were lower than the other measurements. (p < 0.05).

## Conclusions

Dexmedetomidine was superior than the other drugs in preventing laryngoscopy and intubation-related tachycardia. On the other hand, esmolol was superior in preventing systolic, diastolic and mean arterial pressure increases following laryngoscopy and intubation.
